# Symptom Denial and Cultural Constraints: A Qualitative Exploration of Pre‐Hospital Delay Determinants in Myocardial Infarction Patients in China

**DOI:** 10.1002/clc.70343

**Published:** 2026-05-30

**Authors:** Yunjie Tang, Danqing Hu, Jing Han

**Affiliations:** ^1^ School of Nursing Qingdao University Qingdao Shandong China; ^2^ Qingdao Municipal Key Laboratory for Smart Healthcare and Chronic Disease Management Qingdao Shandong China

**Keywords:** delay factors, myocardial infarction, pre‐hospital delay, qualitative study

## Abstract

**Objective:**

To explore the pre‐hospital delay experiences of myocardial infarction patients in China from a patient‐centered perspective.

**Background:**

Pre‐hospital delay remains a globally prevalent issue, influenced by subjective and context‐dependent factors that are not easily captured through quantitative methods. Traditional metrics may oversimplify the psychological processes involved and fail to account for individual variability and contextual influences.

**Design:**

A descriptive qualitative study.

**Methods:**

Semi‐structured interviews were conducted with MI patients at a tertiary hospital in Qingdao between November 18 and 30, 2024. Participants were selected through purposeful sampling. Thematic analysis was performed using MAXQDA software to code and analyze the qualitative data. The study followed the COREQ checklist.

**Findings:**

We interviewed 16 patients and conducted four core themes: (1) limited disease awareness, (2) inappropriate symptom response strategies, (3) constraints of traditional cultural beliefs and (4) family influence on decision‐making, along with 12 subthemes.

**Conclusion:**

Patients' pre‐hospital delays frequently stem from cognitive limitations in symptom recognition coupled with maladaptive coping strategies contributes to treatment postponement. Notably, familial decision‐making intervention demonstrates potential in mitigating such delays. And traditional Chinese culture exerts a profound influence on pre‐hospital delays among elderly MI patients.

**Implications for Patients:**

Patients' experiences of pre‐hospital delay in myocardial infarction reveal its key determinants. Healthcare providers should develop targeted interventions tailored to patients' cognition, culture, and family situations to enhance intervention effectiveness.

**Patient or Public Contribution:**

Patients and family members contributed to the data collected.

## Graphical abstract

This study reveals that heart attack patients often dangerously delay emergency care due to unrecognized symptoms, passive endurance, and traditional cultural constraints, like fearing to burden families. Crucially, family involvement acts as a double‐edged sword, either accelerating or further delaying life‐saving treatment.

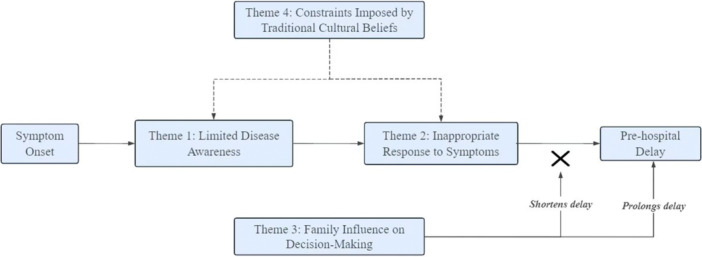

## 1 Introduction

Myocardial infarction (MI) refers to myocardial necrosis caused by acute coronary artery occlusion due to plaque rupture, hemorrhage, or thrombus formation in the context of coronary atherosclerotic stenosis [1]. As a major cause of cardiovascular death, MI poses a serious public health burden. In 2022, there were 1.034 million hospitalizations for acute MI in China, with an in‐hospital mortality rate of 4.3% [2]. Timely revascularization in MI is critical, as each minute of delay is associated with irreversible myocardial damage and increased mortality risk [3]. It has been estimated that for every hour of delay in seeking medical attention, the risk of death increases by 10% [4].

Despite the importance of timely treatment, pre‐hospital delay, defined as the time from symptom onset to arrival at the hospital, remains a globally widespread issue [5, 6, 7, 8]. In China, a large cross‐sectional study involving over 30 000 MI patients [9] found that 70.7% arrived at the hospital more than 2 h after symptom onset, indicating a high incidence of pre‐hospital delay. Understanding the factors contributing to such delays is essential for developing effective interventions to improve outcomes.

Pre‐hospital delay is a multifaceted issue influenced by subjective and context‐dependent factors that are not easily captured by quantitative methods. Traditional metrics may oversimplifying psychological processes and fail to account for individual variability and contextual influences. For instance, a cross‐sectional study [10] examining the impact of health literacy on pre‐hospital delay among MI patients utilized the Brief Health Literacy Screen (BHLS) to assess health literacy. However, this scale not only evaluates patients through only five themes but also lacks unified scoring criteria, thus failing to sufficiently explain the specific influence of health literacy on pre‐hospital delays. Compared with objective factors such as hospital accessibility and transportation [11], qualitative research offers unique insights into patients' psychological, cognitive, and behavioral responses to MI symptoms. Such insights can inform the development of more targeted and patient‐centered intervention. Accordingly, this study aims to explore the lived experiences of MI patients who experienced pre‐hospital delays in Qingdao, China. It adopts a patient‐centered perspective and systematically investigates the contributing factors to support the development of effective strategies for reducing pre‐hospital delays.

## 2 Methods

### 2.1 Study Design

This study is guided by the Common‐Sense Model of Self‐Regulation (CSM) as its theoretical framework. The model, from a cognitive‐behavioral perspective, systematically explains the self‐management processes individuals engage in response to current and potential health threats, emphasizing the dynamic interplay among perception, behavior, and cognition [12]. CSM provides a complete theoretical path for analyzing pre‐hospital delay behavior by dissecting the patients' perception process of health threats (including threat identification, emotional response, and cognitive assessment), the formation mechanism of coping behaviors (planning and execution of action plans), and the continuous adjustment based on feedback effects (reassessment of the efficacy of measures and disease progression). In this study, the authors followed the Consolidated criteria for Reporting Qualitative research (COREQ) checklist [13].

### 2.2 Participants and Sampling

Between November 18 and 30, 2024, we recruited patients who experienced pre‐hospital delay of 2 h or more (from symptom onset to hospital arrival) at a tertiary hospital in Qingdao using purposeful sampling. To ensure sample diversity, participant characteristics were analyzed after each interview to guide subsequent recruitment, continuing until thematic saturation was reached. During the study, 21 potential participants were contacted, and five declined to participate due to time constraints. No repeat interviews were conducted.

### 2.3 Inclusion Criteria

1. Diagnosis according to the 2018 Universal Definition of MI [14];
2. Symptom‐to‐door time ≥ 2 h [5];
3. Age ≥ 18 years;
4. Intact communication capacity;
5. Admission within 72 h of symptom onset.

### 2.4 Exclusion Criteria

1. History of psychiatric disorders;
2. Incomplete symptom or hospital arrival records;
3. Severe comorbidities.

### 2.5 Sample Size

Thematic saturation determined sample adequacy. Recruitment ceased when three consecutive interviewees provided no novel thematic content.

## 3 Study Methods

### 3.1 Confirm the Interview Outline

This study employed a descriptive phenomenological research approach using semi‐structured interviews to collect data. The initial interview guide was developed based on a systematic literature review and its content was validated by two clinical cardiology experts. The final interview guide comprised six key domains: (1) What symptoms did you experience during your MI? (2) Where were you and what were you doing when the symptoms began? (3) At that time, what did you believe these symptoms were related to? (4) What was your first reaction when you felt unwell? (5) Did any bystanders offer you help or advice? What kind of help or advice did they give? (6) What factors contributed to your hesitation in seeking medical treatment?

### 3.2 Data Collection

Ethical approval was granted by the Ethics Committee of Medical College of Qingdao University (QDU‐HEC‐2024414), with written informed consent obtained from all participants.

The interviews were conducted from November 18, 2024 by the first author who had be trained and experienced in conducting qualitative research. A close relationship was established between interviewers and potential participants through close and meticulous care services after hospitalization. To ensure the safety and privacy of the interviewees, while avoiding interference, we conducted interviews in partitioned cubicles within the emergency inpatient ward. The duration of the interview was based on the principle that the interviewees do not feel tired and the vital signs shown by the ECG monitor are stable. Each interview lasted 20–30 min. Before each interview, the interviewer provided a brief self‐introduction, a detailed explanation of the study purpose, procedures, and confidentiality measures, including anonymized data handling and voluntary participation. We obtained written informed consent, with audio recordings strictly reserved for academic use. Interviewers employed active listening, neutral probing, and observational techniques to document verbal and non‐verbal responses.

### 3.3 Data Management and Analysis

We transcribed audio recordings verbatim within 24 h and imported them into MAXQDA 2020 for analysis. We replaced participant identities with alphanumeric codes to ensure anonymity. We conducted analysis according to the thematic analysis method steps [15]:

1. Immersion: Repeated review of transcripts;
2. Initial coding: Line‐by‐line open coding;
3. Theme development: Cluster codes into candidate themes;
4. Theme refinement: Iterative revision via team discussions;
5. Theme definition: Contextualize themes within the CSM framework;
6. Reporting: Synthesize findings with illustrative quotes.

### 3.4 Quality Assurance

To mitigate bias:

1. Interviewers maintained neutrality, avoiding leading questions or judgments;
2. We prioritized patients admitted ≤ 72 h post‐symptom onset to minimize recall bias;
3. We triangulated symptom onset and hospital arrival times with EMS records and chest pain center timestamps;
4. We analyzed transcripts within 24 h of interviews to preserve contextual accuracy;
5. We cross‐verified clinical timelines (e.g., door‐to‐balloon time) via hospital information systems.

## 4 Results

A total of 16 patients (age range: 37–81 years) participated in the study. The cohort was predominantly male (*n* = 13, 81.25%) and largely resided in urban areas (*n* = 9, 56.25%). Regarding educational attainment, participants held a college degree or higher (*n* = 4, 25.0%), a high school diploma (*n* = 2, 12.5%), a middle school certificate (*n* = 5, 31.25%), or primary education and below (*n* = 5, 31.25%). Occupational distribution included employed individuals (*n* = 8, 50.0%), farmers (*n* = 6, 37.5%), and retirees (*n* = 2, 12.5%). Clinically, nine participants (56.25%) had pre‐existing chronic conditions such as hypertension, diabetes, or coronary heart disease, one (6.25%) presented with systemic lupus erythematosus, and six (37.50%) reported no significant medical history. Notably, at the time of symptom onset, the vast majority of participants (*n* = 12, 75.0%) failed to attribute their initial symptoms to a cardiac event (Table 1). Thematic analysis identified four core themes: (1) Limited disease awareness, (2) Inappropriate symptom response strategies, (3) Family influence on decision‐making, and (4) constraints imposed by traditional cultural beliefs. Twelve subthemes further delineated contextual variations within these domains (see Tables 2 and 3).

**TABLE 1:** Basic information of interviewees.

Variables	Number	Frequency (%)
Age		
< 60	11	68.75
≥ 60	5	31.25
Gender		
Male	13	81.25
Female	3	18.75
Habitation		
Urban	9	56.25
Rural	7	43.75
Chronic disease history		
Yes	10	62.50
No	6	37.50
Educational level		
College or above	4	25.00
High school	2	12.50
Middle school	5	31.25
Primary school or below	5	31.25
Occupation		
Employed	8	50.00
Farmer	6	37.50
Retired	2	12.50
Initial symptom		
Chest pain	9	56.25
Chest tightness	4	25.00
Other	3	18.75
First person contacted		
Wife	10	62.50
Husband	2	12.50
Son	3	18.75
None	1	6.25
Transport Mode		
Private car	9	56.25
Ambulance	5	31.25
Taxi cab	1	6.25
Walking	1	6.25
Notice cardiac related		
Yes	4	25.00
No	12	75.00
Onset time of symptom		
Day	9	56.25
Night	7	43.75
Prodromal symptoms		
Yes	11	68.75
No	5	31.25

**TABLE 2:** Themes and subthemes.

Themes	Subthemes
Limited disease awareness	1. Failure to recognize symptom severity 2. Misattribution of atypical symptoms 3. Normalization of prodromal symptoms 4. Habituation to symptom patterns 5. Health overconfidence
Inappropriate responses to symptoms	1. Rest‐based waiting until symptom resolution 2. Self‐medication misuse (1) Pharmacological misdirection (2) Therapeutic complacency
Family influence on decision‐making	1. Professional guidance from healthcare‐affiliated relative 2. Familial advocacy overcoming patient resistance (1) Family‐Coerced Medical Intervention (2) Collaborative Decision‐Making
Constraints imposed by traditional cultural beliefs	1. Delayed care‐seeking until daylight 2. Medical system distrust (1) Therapeutic disillusionment (2) Cultural preference for traditional medicine 3. Familial responsibility paradox (1) Concealment to protect offspring (2) Ageist therapeutic nihilism

**TABLE 3:** Extracting information and naming themes and sub‐themes.

Meaning unit	Descriptions of meanings	Themes	Subthemes
“I felt a sharp pain here (gesturing to chest), like my heart was being squeezed. It had been bothering me for days, but I thought it was just indigestion.” (A10)	The patient failed to recognize myocardial infarction and thought it was just an uncomfortable stomach.	Limited disease awareness	Failure to recognize symptom severity
“The pain struck right in the early hours, around two or three in the morning. At the time, I just told myself to endure it; I figured if I could just bear it until dawn, the pain would pass, and I could get up and go to work.” (A11)	The patient suffered discomfort but chose to endure it until the symptoms disappeared.	Inappropriate responses to symptoms	Rest‐based waiting until symptom resolution
“I called my cousin, a cardiothoracic specialist, during the attack. He urgently instructed me to head to the Emergency Room (ER) immediately. I went straight there while coordinating with him on the way.” (A13)	The patient consulted a family member who is a healthcare worker, sought medical attention promptly, and reduced the delay in treatment.	Family influence on decision‐making	Professional guidance from healthcare‐affiliated relatives
“I kept waiting, thought the pain would subside. It was already quite late, and I didn't want to disturb the family at this hour. I walked to the clinic after daybreak.” (B1)	The patient experienced discomfort at night but, due to the late hour, chose to wait until daytime to seek medical care.	Constraints imposed by traditional cultural beliefs	Delayed care‐Seeking until daylight

## 5 Theme 1: Limited Disease Awareness

### 5.1 Failure to Recognize Symptom Severity

Most participants demonstrated inadequate recognition of MI‐related symptoms. Even those presenting with classic manifestations (e.g., chest tightness, substernal pain) often misinterpreted their significance. One participant recounted: “I felt a sharp pain [here‐gesturing to chest], like my heart was being squeezed. It had been bothering me for days, but I thought it was just indigestion.” (A10) Another participant described a similar failure to recognize the Symptom Severity: “At that time, the pain was a tingling sensation, and it gradually intensified. It started from the navel and became more and more painful, just like someone using a hammer to strike my chest. I thought it was hunger, but I didn't go to eat. I thought the hunger would subside after a while, so I continued working after feeling dizzy.” (A4)

### 5.2 Misattribution of Atypical Symptoms

Patients presenting with non‐classical manifestations—such as epigastric discomfort, nausea, or radiating back pain—exhibited a significantly greater tendency to dismiss their symptoms. These atypical presentations were frequently misattributed to benign, non‐cardiac conditions, thereby substantially impairing patients' perception of cardiac risk. One participant illustrated this musculoskeletal misattribution: “I assumed it was a pulled muscle and tried massage therapy. The dull ache persisted, but I didn't connect it to heart issues.” (A6) Similarly, gastrointestinal symptoms were commonly dismissed as minor dietary disturbances, as another patient reported: “I thought I had just eaten something bad that morning; it never crossed my mind that it could be related to my heart.” (A8) Such erroneous causal attributions led to inappropriate self‐management strategies, critically prolonging pre‐hospital delay.

### 5.3 Normalization of Prodromal Symptoms

More than half of the participants (*n* = 11/16) reported experiencing prodromal manifestations, such as recurrent chest discomfort, for up to a month preceding the acute MI onset. Most chronically dismissed these warning signs, resorting to passive waiting rather than seeking clinical evaluation. Symptom resolution was often misinterpreted as spontaneous recovery, fostering a dangerous cognitive habituation to the discomfort. One participant illustrated this psychological normalization: “I didn't have any major reaction because I had experienced this kind of situation before; previously, it would just gradually subside.” (A9) Similarly, the rapid but temporary relief of symptoms validated passive coping mechanisms for others: “I had experienced this exact situation before; previously, the discomfort would just subside after I rested for 3 or 4 min.” (A7) Furthermore, gastrointestinal misattribution frequently masked these critical warning signs and severely delayed care‐seeking: “I've had this problem since about a month ago. Later on, it became a frequent ache. At first, I thought it was a stomachache; I assumed the pain was coming from my digestive organs.” (A4)

### 5.4 Habituation to Symptom Patterns

Patients repeatedly suffer from chest pain maybe cultivated dangerous habituation (e.g., self‐medication). Participants frequently applied historical coping strategies to acute events, erroneously anticipating spontaneous resolution. A participant explained: “I'd had similar mild episodes before—they always faded in 3–4 min. When this severe pain hit, I kept waiting, assuming it would pass like before.” (A7) This behavioral inertia, rooted in prior “successful” symptom management, critically exacerbated treatment delays. Another participant echoed this reliance on past experiences: “At that time, I was working and going to work. I didn't have any major reaction because I had experienced this kind of situation before [referring to chest tightness and discomfort in the chest]. However, before that, it would gradually get better.” (A9)

### 5.5 Health Overconfidence

Middle‐aged and young MI patients commonly exhibited health perception biases, manifested in two psychological patterns. Both groups expressed disbelief post‐diagnosis, a response potentially linked to self‐image preservation efforts that suppressed their healthcare‐seeking intentions.

#### 5.5.1 Age‐Related Denial

Unfounded confidence in youth or past health triggered strong psychological denial. Confronted with sudden symptoms, these patients could not reconcile the illness with their established “healthy” self‐image. For instance, a 44‐year‐old participant stated: “I never imagined having heart issues at my age.” (A8) Similarly, a 59‐year‐old farmer reflected: “I initially underestimated the severity, planning to visit outpatient clinics gradually. I previously engaged in heavy alcohol consumption and excessive smoking but recently quit. My prior robust health and age made cardiac problems inconceivable.” (A4) This overconfidence prevented them from taking the symptoms seriously and seeking immediate help.

#### 5.5.2 False Reassurance From Checkups

Some participants appeared to develop a false sense of security due to previous normal checkup results. Consequently, upon the onset of acute symptoms, these patients tended to misjudge their condition, sometimes attributing severe warning signs to non‐cardiac issues. One participant stated: “My routine checkups consistently showed no abnormalities, so I always considered myself in excellent health and seldom sought medical care. I never anticipated developing such a severe condition.” (A13) Similarly, another patient's reliance on past examinations delayed their recognition of the current threat: “I know the director of a local hospital, and I often go there for physical examinations. I even had an electrocardiogram done before, and the results showed that I was completely normal.” (A5) This suggests that the cognitive inertia shaped by a history of normal checkups may contribute to inappropriate coping behaviors, such as symptom denial and delayed care‐seeking.

## 6 Theme 2: Inappropriate Response to Symptoms

### 6.1 Rest‐Based Waiting Until Symptom Resolution

Some participants adopted passive endurance as their primary coping mechanism, erroneously equating symptom tolerability with a non‐critical status. This maladaptive “wait‐and‐see” behavior significantly delayed medical care until the symptoms became completely unbearable. One participant described: “The pain struck right in the early hours, around two or three in the morning. At the time, I just told myself to endure it; I figured if I could just bear it until dawn, the pain would pass, and I could get up and go to work.” (A11) Furthermore, transient symptom palliation often reinforced this passive waiting. Another patient recounted: “Since we were over in Jiaozhou, we initially thought about heading to the emergency department. But after enduring it and resting for a while, I felt a bit better. So, we simply waited until daytime to register for a regular outpatient appointment.” (B2) Such delayed help‐seeking, driven by normalized pain thresholds and the misinterpretation of temporary relief, severely exacerbated pre‐hospital delays during the critical golden window for reperfusion.

### 6.2 Self‐Medication Misuse

#### 6.2.1 Pharmacological Misdirection

Some participants inappropriately self‐medicated to manage their acute symptoms, falsely attributing atypical systemic or referred discomfort to non‐cardiac ailments. This maladaptive coping strategy created a dangerous false sense of security, which prematurely terminated their care‐seeking trajectory. For instance, gastrointestinal misattribution led one participant to rely on inappropriate remedies: “I lay in my car taking stomach pills, convinced it was indigestion, just waiting for the medication to take effect.” (A8) Similarly, atypical presentations were frequently misinterpreted as common infections, prompting the misuse of over‐the‐counter cold medications or antibiotics. Another patient recounted how this infectious misattribution severely delayed emergency care: “After that, I felt a bit of pain and coldness. I thought it was a cold or a fever, so I went home and took some medicine. It seemed to be a fever‐reducing pill. After the fever went down, I had some food. I took the cold medicine and also took Cefalexin.” (A11)

#### 6.2.2 Therapeutic Complacency

Even when utilizing appropriate cardiac‐specific medications (e.g., nitroglycerin or rapid‐acting traditional remedies), patients frequently misinterpreted transient symptom palliation as definitive disease resolution. This false sense of security prematurely terminated their self‐regulation and care‐seeking processes. One participant described how repeated, temporary relief from a traditional cardiac medication fostered a dangerous habituation to the warning signs: “I had experienced discomfort several times before. My wife would give me Jiuxin pills [a rapid‐acting traditional cardiac medication]. At those times, the pain radiated through my chest and back, but after taking the medicine and stretching my arms a bit, the pain would ease, so I simply ignored it.” (A10)

Similarly, this temporary pharmacological palliation critically postponed definitive care even during severe acute episodes. Another case illustrated how immediate but transient relief disrupted emergency decision‐making during a nocturnal attack: “Waking up choking, I took Jiuxin pills and felt better immediately. I assumed the crisis was over—until the pain returned uncontrollably hours later.” (B3)

## 7 Theme 3: Family Influence on Decision‐Making

### 7.1 Professional Guidance From Healthcare‐Affiliated Relatives

Patients who sought assistance from relatives or friends with medical backgrounds benefited from prompt professional guidance. This crucial intervention helped them accurately assess the severity of their condition and receive immediate, actionable directives, effectively mitigating pre‐hospital delays caused by indecisiveness. One participant described how consulting a specialist relative streamlined his response: “I called my cousin, a cardiothoracic specialist, during the attack. He urgently instructed me to head to the Emergency Room (ER) immediately. I went straight there while coordinating with him on the way.” (A13) Similarly, another patient benefited immensely from his spouse's clinical literacy: “I told my wife that I was feeling unwell. She immediately replied, ‘Then let's go to the hospital.’ There was no hesitation on her part because she has a nursing background and quickly recognized that it was heart‐related. Moreover, my shoulder was also aching that day, presenting as a classic radiating pain.” (A2)

### 7.2 Familial Advocacy Overcoming Patient Resistance

Family members played pivotal roles in counteracting patients' denial/minimization tendencies. Two intervention patterns emerged:

#### 7.2.1 Family‐Coerced Medical Intervention

When patients formed benign illness representations and employed maladaptive coping strategies (i.e., symptom denial) to resist medical care, spouses or family members frequently acted as a crucial external regulatory force. By making a more accurate threat appraisal, they intervened forcefully, overriding the patient's reluctance and insisting on immediate hospitalization. One participant admitted how his spouse's decisiveness countered his own dismissal of the symptoms: “I refused to go, blaming exhaustion. But my wife insisted: ‘This isn't normal—we're going NOW.’ Her urgency overrode my denial.” (A11) Similarly, another patient recounted how his wife bypassed his financial concerns and took decisive emergency action: “The pain woke me up at night, and it was extremely uncomfortable. I just asked my wife for some medicine. She quickly found a 'heart‐protecting' pill [a traditional rapid‐acting cardiac medication] for me and then immediately dialed EMS. Honestly, I didn't want to go because I was worried about the medical costs. But seeing the profuse sweat on my body, she ignored my protests and insisted that I must go to the hospital.” (A10)

#### 7.2.2 Collaborative Decision‐Making

Our interview data reveals a strong reliance on family support, with the vast majority of participants (*n* = 15/16) choosing to disclose their symptoms to family members before seeking medical help. This initial sharing of symptoms facilitated a collaborative assessment of the emergency. When patients communicated their discomfort, family members were often able to help contextualize the severity of the situation, particularly when visible signs such as extreme pallor were present. By strongly urging the patient to seek immediate medical attention, family members, especially spouses, played a critical role in overcoming the patient's hesitation and driving the final decision to visit the emergency department. One participant illustrated how his wife's observation of his severe physical signs led to immediate intervention: “I told my wife I felt unwell and was going to sleep for a bit. When she came back to check on me, she saw my face had turned a sickly, ashen‐yellow color, and I was in no condition to drive. She immediately got a relative to drive us straight to the hospital.” (A8) Another patient highlighted the crucial role of his spouse's urging compared to the bystander effect of coworkers: “I didn't tell my coworkers because it wouldn't have been any use. Instead, I told my wife. She strongly urged me to hurry to the hospital, so I immediately went and registered at the emergency department.” (A9)

## 8 Theme 4: Constraints Imposed by Traditional Cultural Beliefs

### 8.1 Delayed Care‐Seeking Until Daylight

Elderly participants experiencing nocturnal symptom onset frequently postponed seeking medical care until daylight, enduring discomfort due to concerns about disturbing family members, long travel distances to medical facilities, or nocturnal transportation challenges:

“I kept waiting, thought the pain would subside. It was already quite late, and I didn't want to disturb the family at this hour. I walked to the clinic after daybreak.” (B1)

“At 2 a.m., enduring seemed wiser than burdening my family with a nighttime hospital trip.” (A11)

### 8.2 Medical System Distrust

Two distrust pathways emerged:

#### 8.2.1 Therapeutic Disillusionment

Negative prior medical encounters, whether experienced personally or vicariously through family members, created a strong distrust in the healthcare system among patients. These experiences made them highly reluctant to seek hospital care, significantly influencing their decision‐making during acute episodes. One participant said: “I went to Chengyang Hospital for a check‐up before, but they didn't find anything. The hospital suggested 24‐h monitoring, but I felt that once that brief episode passed, I was completely fine—just like nothing had happened. Therefore, I basically never see a doctor unless I absolutely can't endure it anymore.” (A10) This cognitive resistance was clearly articulated by another patient, who feared treatment‐induced harm: “My father had heart disease, and I took him to the hospital for over 10 years. He had three stents put in. He was doing quite well before the procedure, but his condition deteriorated significantly afterward. He even struggled to get out of bed after the stents. Because of that, I strongly resist these kinds of treatments.” (A9)

#### 8.2.2 Cultural Preference for Traditional Medicine

Influenced by deep‐rooted cultural beliefs, some participants exhibited a strong predilection for Traditional Chinese Medicine (TCM), prioritizing herbal remedies over conventional hospital care during symptom onset. This perceived superiority of TCM often led them to dismiss or delay seeking Western medical evaluation. One participant expressed a fundamental distrust of conventional medicine: “Herbal regimens failed, but I still distrust Western drugs—they're temporary fixes, not cures.” (B1) Another participant described how relying on TCM delayed critical care for over a week: “About 10 days ago, I felt a bit unwell. I went to see a traditional Chinese doctor and took some medicine. However, the discomfort didn't subside over those 10 days. This dragged on right up until that weekend morning [referring to the day of the severe myocardial infarction and admission].” (A3)

### 8.3 Familial Responsibility Paradox

#### 8.3.1 Concealment to Protect Offspring

Elderly participants, driven by a strong sense of familial responsibility, intentionally concealed their physical ailments to avoid burdening their adult children. Practical concerns over exorbitant medical costs, occupational disruptions, and childcare obligations for grandchildren frequently motivated this intentional symptom concealment, ultimately contributing to delayed care‐seeking. One participant expressed this self‐sacrificing mindset clearly: “My son's family juggles careers and grandchildren. Burdening them with my health felt selfish.” (B3) Another participant shared how this desire to avoid causing trouble led to prolonged pre‐hospital delay: “I actually had this symptom—my heart had been feeling uncomfortable for about 2 months. But I hadn't told my son about it. I just wanted to endure the minor illness for a while, as I didn't want to cause any trouble for him. I didn't expect that by enduring it for so long, I would end up with a serious illness.” (B2)

#### 8.3.2 Ageist Therapeutic Nihilism

Elderly participants' fatalistic attitudes toward aging often rationalized their care refusal. This self‐sacrificial ethos—equating illness disclosure with familial disruption—transformed care‐seeking into a perceived moral failure. For instance, one participant expressed a reluctance to burden their family financially: “At 72, I've lived fully. Why drain my children's savings for uncertain treatments?” (B1) This sense of resignation was echoed by another patient, who viewed physical decline as an inevitable reason to give up: “Both my wife and I often suffer from these diseases, so we didn't take them seriously. As I got older, I had to be hospitalized for long periods of time. My body would have problems here or there, so there was no point in continuing.” (B3) Consequently, this fatalistic normalization of suffering led them to passively endure symptoms rather than seek life‐saving interventions.

## 9 Discussion

This study adopts the Common‐Sense Model of Self‐Regulation as its theoretical framework to explore myocardial infarction patients' pre‐hospital delay experiences from their perspective by analyzing the cognitive‐behavioral processes involved in threat perception and behavioral responses. The investigation identifies the latent contributing factors and underlying treatment behind treatment‐seeking delays, leading to the extraction of four principal themes: limited disease awareness, inappropriate symptom response strategies, family influence on decision‐making, and constraints imposed by traditional cultural beliefs. These findings provide valuable insights and preliminary evidence for developing targeted interventions to reduce pre‐hospital delays in myocardial infarction management.

This study identified patients' limited cognition of myocardial infarction as a primary contributor to pre‐hospital delays, aligning with findings from previous research. For example, Nadja's survey [16] of over 600 participants across four German federal states demonstrated that individuals with greater knowledge of myocardial infarction were more likely to respond promptly during acute episodes. Similarly, Jin's meta‐analysis [17] concluded that patients' difficulty in recognizing acute myocardial infarction symptoms is a key factor contributing to prolonged pre‐hospital delays. However, prior studies have often oversimplified this cognitive limitation as a mere lack of knowledge. For instance, Yin's qualitative study [18] superficially attributed patients misinterpreting symptoms as non‐cardiac conditions (e.g., bronchitis or stomach pain) to inadequate insufficient myocardial infarction knowledge, without further exploration of the underlying treatment.

Notably, our study revealed a critical paradox: several patients who had received prior education on myocardial infarction still experienced significant pre‐hospital delays. As one participant shared, “I only realized this was a heart attack after I was diagnosed,” (A9) highlighting a profound disconnect between theoretical knowledge of symptoms and real‐world experiential recognition. These observations suggest that while improving knowledge of myocardial infarction may help reduce delays, knowledge dissemination alone is insufficient to significantly improve pre‐hospital response times [19]. Unlike previous studies, our research further identifies four specific manifestations of delays associated with cognitive limitations: failure to recognize the severity of symptoms, misattribution of atypical symptoms, ignoring abnormal sensations, and a self‐perception of being in good health. These findings suggest that pre‐hospital delay is not merely a passive consequence of ignorance, but rather an active psychological process. This process is frequently driven by psychological denial (e.g., overconfidence in youth), misinterpretation of atypical symptoms, and deep‐rooted cultural pressures. Capturing these internal psychological struggles helps explain why even patients who received prior heart disease education may still hesitate during actual emergencies, as their decisions involve complex challenges in symptom interpretation and risk assessment. Therefore, interventions should not only focus on improving knowledge but also target the deeper cognitive biases.

Many myocardial infarction patients engage in self‐treatment behaviors before seeking hospital care [17], hoping to alleviate their symptoms. In this study, many respondents reported that when they first felt unwell, they chose to rest and wait for the symptoms to subside on their own. Atypical symptoms such as toothache, stomachache, and shoulder or back pain often lead patients to underestimate the seriousness of their condition, prompting conventional self‐care measures. For example, some participants described that after feeling radiating pain in the left shoulder, they took a series of measures to relieve muscle damage, such as massage and hot compress; however, they only sought hospital care when symptoms worsened beyond tolerance, resulting in significantly prolonged pre‐hospital delay. Besides rest, self‐medication was a common strategy. Misunderstandings about the use of nitrates and painkillers were widespread. Several patients mistakenly believed that their condition had improved after taking nitrates (such as nitroglycerin) or painkillers when their symptoms temporarily subside, and refused further medical examinations, significantly increasing the risk of adverse outcomes. In addition, persistent symptoms from chronic diseases sometimes caused patients to become desensitized to pain and neglect timely treatment [20]. These findings highlight the necessity of correcting harmful self‐treatment practices through interventions. It is essential to emphasize that self‐assessment cannot replace professional medical judgment and to promote appropriate response strategies, especially stressing the importance of immediate hospital care even after symptom relief.

Elderly patients often exhibit a less sensitive response to pain due to the decline in physical function [21] and the presence of chronic illnesses, which contribute to prolonged pre‐hospital delays [22, 23]. Beyond these physiological factors, social and cultural influences also play a significant role in shaping patients' healthcare behaviors. For example, a study conducted within an Islamic community found that pre‐hospital delays among myocardial infarction patients increased significantly during the traditional Ramadan festival [24]. Similarly, immigrants facing cultural adaptation challenges tend to experience longer pre‐hospital delays. In this study, several elderly respondents exhibited reluctance to seek hospital care because they did not want to disturb their children or had a fatalistic attitude. Some chose to endure the symptoms at night until the next day to avoid disturbing their children's rest and work, resulting in longer pre‐hospital delay. This suggests that traditional cultural factors, deeply embedded in social contexts, influence elderly patients' decision‐making regarding timely medical attention.

Specifically, in Shandong, known as the birthplace of Confucian culture, the elderly population is strongly influenced by Confucian values. The ethical principle of “filial piety” promoted by Confucianism makes the elderly prioritize avoiding the trouble of going to the hospital over their own health. Consequently, when they feel unwell, they tend to endure discomfort rather than seek prompt medical care, thereby contributing to pre‐hospital delays. A survey of 3000 elderly people in Shandong [25] showed that patients in Shandong often have a decision‐making delay of about 1 h before deciding to be admitted to the hospital. Therefore, interventions aimed at reducing pre‐hospital delay should consider the cultural and family backgrounds of elderly patients. Family members should be engaged to help correct misconceptions shaped by traditional culture, such as avoiding medical treatment or not wanting to burden their children. Effective communication with elderly patients about the benefits of timely medical treatment for themselves and their families is crucial to promote timely hospital visits.

Making timely and appropriate decisions when experiencing discomfort can help reduce pre‐hospital delay and improve prognosis. However, in many cases, the decision to seek hospital care is made jointly with family members. Many respondents in the study reported consulting their spouses when feeling unwell, and after reaching a family consensus, proceeding directly to the hospital. This family decision‐making process acts as a double‐edged sword in myocardial infarction pre‐hospital delay. On one hand, family involvement can reduce misjudgment caused by individual perception bias and enhance decision‐making efficiency, thereby preventing pre‐hospital delay due to individual indecision. When patients are reluctant to seek medical help, family members often play a crucial role in encouraging or even insisting on hospital visits, such as spouses urging their partners, children accompanying or forcing their parents to seek care, or directly calling emergency services, all of which help shorten pre‐hospital delay. On the other hand, patients' concerns about family responsibilities, combined with differing reactions among family members to health changes, can make decision‐making more complex and delay timely hospital visits [26]. When patients prioritize concern about “family well‐being” over their own health risk, they may conceal their symptoms to avoid burdening others or minimize the severity of symptoms that could cause family anxiety (e.g., chest pain). Such behavior can hinder reaching family consensus and increase the time needed to decide on hospital care. Therefore, interventions should target not only the patients but also their families. Enhancing family members' awareness and understanding of myocardial infarction symptoms and the urgency of timely treatment is essential to facilitate faster, more effective decision‐making. At the same time, it is important to emphasize that the benefits of early treatment outweigh those of concealing illness, and that making timely, appropriate decisions is a responsibility to the family.

Targeted interventions can be stratified by age with distinct priorities and tailored delivery formats. For young patients, interventions should focus on mitigating “health overconfidence” and correcting the misconception that myocardial infarction is exclusively a disease of the elderly [27]. For middle‐aged patients, strategies should reinforce the concept that maintaining one's own health is the primary prerequisite for caring for family, thereby promoting timely medical evaluation to reduce pre‐hospital delay [28]. For older adults, targeted interventions could adopt highly accessible formats, such as easy‐to‐understand short videos, attempting to help mitigate cultural constraints, particularly the feelings of guilt associated with their illness or the fear of becoming a burden to their children [29].

## 10 Limitations

This study has several limitations. First, participants were recruited from a single tertiary hospital in Qingdao, China, which may limit the transferability of our findings to other populations. Second, limiting the interview duration to 20–30 min may constrain deep exploration of patients' psychological experiences related to pre‐hospital delay, but to minimize recall bias, interviews were conducted within 72 h of admission, and this 20–30 min duration was adopted based on clinical recommendations to reduce patients' physical burden. Third, although our findings indicate that family involvement has a dual impact on pre‐hospital delay, our study design and purposive sampling strategy did not specifically address family structural differences, and thus we were unable to further explore variations in decision‐making efficiency and key influencers across diverse family structures (e.g., elderly individuals living alone, nuclear families, or multi‐generational households). Furthermore, while our interview guide explored patients' symptom contexts, attribution, and reasons for hesitation, it did not specifically inquire about their sources of myocardial infarction‐related information (particularly emerging digital platforms such as short‐form videos), limiting our ability to assess whether such media might help mitigate insufficient disease awareness and related cultural constraints.

## 11 Conclusion

This study highlights key contributors to pre‐hospital delay in myocardial infarction, including cognitive limitations, coping behaviors, sociocultural beliefs, and family decision‐making. Effective interventions should focus on addressing cognitive biases and involving family members to facilitate timely decisions. Considering patients' social and cultural contexts is crucial for reducing delays and improving outcomes.

### 11.1 Relevance to Clinical Practice

The findings of this study provide valuable insights for healthcare professionals and community hospitals in designing and implementing interventions for older adults at risk of myocardial infarction.

It is essential to deliver targeted guidance on symptom recognition and emergency response based on the level of disease awareness among high‐risk patients and their families. This should include education on identifying symptoms during acute episodes, as well as instruction on the appropriate use of emergency medications like nitroglycerin, including timing and administration. Particular emphasis should be placed on recognizing atypical symptoms, such as toothache, back pain, or nausea. Simultaneously, family‐centered interventions must be strengthened to improve overall emergency preparedness and collective decision‐making, thereby reducing treatment delays caused by patients' hesitation or misjudgment.

For elderly patients, health education should aim to correct traditional cultural misconceptions, such as believing that symptoms will resolve on their own, excessive faith in traditional Chinese medicine or avoiding medical care to prevent burdening their children. It is important to promote health awareness and encourage appropriate help‐seeking behaviors grounded in the principles of early identification, early seeking help, and early treatment.

### 11.2 What Does This Paper Contribute to the Wider Global Clinical Community

The cognitive limitations contributing to pre‐hospital delay may not be simply due to a lack of knowledge but rather involve more complex challenges in symptom interpretation and risk assessment. Therefore, interventions should not only focus on improving knowledge but also target the deeper cognitive biases.

Elderly patients with myocardial infarction experience prolonged per‐hospital delays due to concerns about burdening their families, overreliance on traditional Chinese medicine, or perceived futility of treatment based on age. Consequently, interventions should include targeted education to identify and address these misconceptions.

## Author Contributions

Yunjie Tang conceived the concept, design, clinical studies, experimental studies, data acquisition, data analysis, statistical analysis, manuscript preparation, manuscript editing. Danqing Hu and Jing Han performed the data analysis, statistical analysis, manuscript editing, manuscript review and take responsibility for the integrity of the work as a whole from inception to published article. Danqing Hu is the statistician.

## 1 Ethics Statement

Ethics approval for this study was obtained from the Ethics Committee of Medical College of Qingdao University with approval number QDU‐HEC‐2024414.

## 2 Conflicts of Interest

The authors declare no conflicts of interest.

## Supporting information

Supporting File 1

## Data Availability

The data that support the findings of this study are available from the corresponding author upon reasonable request.
