# Supplementary material for: Symptom Denial and Cultural Constraints: A Qualitative Exploration of Pre‐Hospital Delay Determinants in Myocardial Infarction Patients in China
Source: Clin Cardiol. 2026 May 30;49(6):e70343. doi: 10.1002/clc.70343 (PMC13239782; doi:10.1002/clc.70343)
Supplement: Supplementary file 1 — Supporting File 1 [file CLC-49-e70343-s001.docx]

Consolidated criteria for reporting qualitative studies (COREQ) checklist for qualitative studies

| No Item | Guide questions/description | Reported on Page |
| --- | --- | --- |
| Domain 1: Research team and reflexivity | | |
| *Personal Characteristics* | | |
| 1.Interviewer/facilitator | Which author/s conducted the interview or focus group? | P3 Data collection |
| 2. Credentials | What were the researcher’s credentials? E.g. PhD, MD | Title Page |
| 3. Occupation | What was their occupation at the time of the study? | Title Page |
| 4. Gender | Was the researcher male or female | Title Page |
| 5. Experience and training | What experience or training did the researcher have? | P3 Data collection |
| *Relationship with participants* | | |
| 6. Relationship established | Was a relationship established prior to study commencement? | P3 Data collection |
| 7. Participant knowledge of the interviewer | What did the participants know about the researcher? e.g. personal goals, reasons for doing the research | P3 Data collection |
| 8. Interviewer characteristic | What characteristics were reported about the interviewer/facilitator? e.g. Bias, assumptions, reasons and interests in the research topic | P3 Data collection |
| Domain 2: study design | | |
| *Theoretical framework* | | |
| 9. Methodological orientation and Theory | What methodological orientation was stated to underpin the study? e.g. grounded theory, discourse analysis, ethnography, phenomenology, content analysis | P2 Study Design |
| *Participant selection* | | |
| 10. Sampling | How were participants selected? e.g. purposive, convenience, consecutive, snowball | P2 Participants and Sampling |
| 11. Method of approach | How were participants approached? e.g. face-to-face, telephone, mail, email | P2 Participants and Sampling |
| 12. Sample size | How many participants were in the study? | P4 Results |
| 13. Non-participation | How many people refused to participate or dropped out? Reasons? | P2 Participants and Sampling |
| *Setting* | | |
| 14. Setting of data collection | Where was the data collected? e.g.  home, clinic, workplace | P3 Data collection |
| 15. Presence of non-participants | Was anyone else present besides the  participants and researchers? | P3 Data collection |
| 16. Description of sample | What are the important characteristics of the sample? e.g. demographic data, date | Table 1 |
| *Data collection* | | |
| 17. Interview guide | Were questions, prompts, guides provided by the authors? Was it pilot  tested? | P3 Data collection |
| 18. Repeat interviews | Were repeat inter views carried out? If yes, how many | NO |
| 19. Audio/visual recording | Did the research use audio or visual  recording to collect the data? | Audio recording  P3 Data Management and Analysis |
| 20. Field notes | Were field notes made during and/or  after the interview or focus group? | During the interview |
| 21. Duration | What was the duration of the inter  views or focus group? | P3 Data collection |
| 22. Data saturation | Was data saturation discussed? | P3 Data collection |
| 23. Transcripts returned | Were transcripts returned to  participants for comment and/or correction? | n/a |
| Domain 3: analysis and findings | | |
| *Data analysis* | | |
| 24. Number of data  coders | How many data coders coded the  data? | P4 Results |
| 25. Description of the  coding tree | Did authors provide a description of  the coding tree? | Table 3 |
| 26. Derivation of themes | Were themes identified in advance or  derived from the data? | P4-P7  Table 3 |
| 27. Software | What software, if applicable, was used to manage the data? | MAXQDA 2020 |
| 28. Participant checking | Did participants provide feedback on  the findings? | n/a |
| *Reporting* | | |
| 29. Quotations presented | Were participant quotations presented to illustrate the themes/findings? Was each quotation identified? e.g.  participant number | Yes  P4-P7 |
| 30. Data and findings  consistent | Was there consistency between the  data presented and the findings? | Yes  P4-P7 |
| 31. Clarity of major  themes | Were major themes clearly presented  in the findings? | Yes  P4 Results |
| 32. Clarity of minor  themes | Is there a description of diverse cases  or discussion of minor themes? | Yes  P4-P7 |

Tong A, Sainsbury P, Craig J. Consolidated criteria for reporting qualitative research (COREQ): a 32-item checklist for interviews and focus groups. *International Journal for Quality in Health Care* 2007;19(6):349 – 357
